# The CpxR regulates type VI secretion system 2 expression and facilitates the interbacterial competition activity and virulence of avian pathogenic *Escherichia coli*

**DOI:** 10.1186/s13567-019-0658-7

**Published:** 2019-05-24

**Authors:** Zhengfei Yi, Dong Wang, Suhua Xin, Dongliang Zhou, Tao Li, Mingxing Tian, Jingjing Qi, Chan Ding, Shaohui Wang, Shengqing Yu

**Affiliations:** Shanghai Veterinary Research Institute, Chinese Academy of Agricultural Sciences, Shanghai, 200241 China

## Abstract

Systemic infections caused by avian pathogenic *Escherichia coli* (APEC) are economically devastating to poultry industries worldwide and are also potentially threatening to human health. Pathogens must be able to precisely modulate gene expression to facilitate their survival and the successful infection. The Cpx two-component signal transduction system (TCS) regulates surface structure assembly and virulence factors implicated in Gram-negative bacterial pathogenesis. However, the roles of the Cpx TCS in bacterial fitness and pathogenesis during APEC infection are not completely understood. Here, we show that the Cpx TCS response regulator CpxR is critical to the survival and virulence of APEC. Inactivation of *cpxR* leads to significant defects in the interbacterial competition activity, invasion and survival of APEC in vitro and in vivo. Moreover, activation of CpxR positive regulates the expression of the APEC type VI secretion system 2 (T6SS2). Further investigations revealed that phosphorylated CpxR directly bound to the T6SS2 *hcp2B* promoter region. Taken together, our results demonstrated that CpxR contributes to the pathogensis of APEC at least through directly regulating the expression and function of T6SS2. This study broadens understanding of the regulatory effect of Cpx TCS, thus elucidating the mechanisms through which Cpx TCS involved in bacterial virulence.

**Electronic supplementary material:**

The online version of this article (10.1186/s13567-019-0658-7) contains supplementary material, which is available to authorized users.

## Introduction

Systemic infections caused by avian pathogenic *Escherichia coli* (APEC) are economically devastating to poultry industries worldwide. APEC shares a broad range of virulence factors with human extraintestinal pathogenic *E. coli* (ExPEC), including uropathogenic *E. coli* (UPEC) and neonatal meningitis *E. coli* (NMEC). Moreover, these ExPEC strains can cause cross infections, thus indicating that APEC may be a potential virulence gene reservoir for UPEC and NMEC [1–4]. APEC initially infects poultry via the respiratory tract, then spreads systemically throughout the entire body. Pathogens use several common strategies to increase fitness and facilitate survival and systemic infections [5, 6]. The conserved type VI secretion system (T6SS), present in more than one-fourth of Gram-negative pathogens, is used to deliver effector proteins into eukaryotic or prokaryotic cells. These effectors have roles in a broad variety of functions, including interbacterial competition, stress sensing, biofilm formation and virulence [7–13]. Three distinct T6SSs have been identified in APEC genomes. Among them, the APEC T6SS2, similar to NMEC T6SS, is responsible for the binding and invasion to host cells, survival, interbacterial competition and pathogenesis of APEC and NMEC [11, 14–16]. As contact-dependent pathways, the T6SSs are tightly regulated by various regulatory mechanisms to ensure successful bacterial infection [17].

When detecting new environmental cues or stresses, bacteria must precisely modulate gene expression to facilitate their survival through complex regulatory networks. The two-component signal transduction systems (TCSs) consist of a histidine kinase sensor in the bacterial plasma membrane and a cytoplasmic response regulator that allows bacteria to cope with changes in the environment [18, 19]. The Cpx TCS of *E. coli* consists of three proteins, CpxA, CpxR, and CpxP, which mainly mediate the detection of and adaptation to envelope stresses [20, 21]. Many environmental cues leading to Cpx TCS activation have been identified, including envelope protein misfolding, overexpression of the pilus and alkaline pH. In addition, adhesion to hydrophobic surfaces activates the Cpx TCS in an NlpE-dependent manner [22–24]. After an environmental signal is received, the kinase histidine CpxA undergoes autophosphorylation and phosphorylates the response regulator CpxR. Then phosphorylated CpxR binds its regulon and functions as a transcriptional regulator. The third component of the Cpx system is CpxP, a small periplasmic protein thought to negatively regulate the Cpx TCS by binding CpxA and maintaining it in an inactive state [23–25].

The Cpx TCS affects bacterial virulence through regulating the expression of virulence genes involved in envelope stress relief, biofilm formation, adherence, motility and secretion systems (type III, type IV and type VI) [26–36]. The activation of Cpx TCS in *Shigella sonnei*, *Legionella pneumophila*, *Xenorhabdus nematophila* and *Yersinia pestis* results in increased expression of virulence genes and contributes to pathogenesis [26, 30, 32, 33, 37, 38]. The Cpx TCS also regulates T6SS expression, and affects the virulence of *Citrobacter rodentium* [31, 36]. However, constitutive activation of the Cpx TCS results in downregulation of virulence genes and attenuated virulence of *Haemophilus ducreyi* [39, 40]. In pathogenic *E. coli*, the Cpx TCS has been implicated in the regulation of pili and the type III secretion system and is responsible for virulence [27, 28, 41–44]. The Cpx TCS has been shown to be involved in the adherence, invasiveness and biofilm formation of APEC through controlling the orientation of the type 1 fimbriae OFF–ON switch. However, the roles of Cpx TCS in the fitness and pathogenesis of APEC during in vivo infection are not completely understood. In this study, we demonstrated that *cpxR* deletion leads to substantial defects in interbacterial competition activity, invasion and survival, and attenuates the virulence of APEC in vitro and in vivo. Moreover, we provide evidence that CpxR positively regulates the expression and function of APEC T6SS2, which may contribute to the systemic infection and pathogenesis of APEC. This study broadens understanding of the regulatory effect of Cpx TCS, thereby elucidating the mechanisms through which Cpx TCS contributes to virulence.

## Materials and methods

### Bacterial strains, plasmids and growth conditions

The strains and plasmids used in this study are listed in Table 1. The APEC strain DE719 (O2:K1) causes severe colibacillosis symptoms and high mortality in ducks and mice [14]. Bacteria were grown routinely in Luria–Bertani (LB) medium at 37 °C with aeration. When necessary, the antibiotic ampicillin (Amp; 100 μg/mL) or chloramphenicol (Cm; 30 μg/mL) was supplemented in the medium.

**Table 1:** **Bacterial strains and plasmids used in this study**

Strains or plasmids	Characteristics	References
Strains		
DE719	O2:K1	[14]
ΔcpxR	*cpxR* gene deletion mutant in DE719	This study
CΔcpxR	ΔcpxR with plasmid pSTV28-cpxR	This study
DH5α-pRCL	DH5α with plasmid pRCL	This study
DH5α-pRCL-P_*hcp2B*_	DH5α with plasmid pRCL-P_*hcp2B*_	This study
DE719-pBAD	DE719 with plasmid pBAD/Myc-His	This study
DE719-pnlpE	DE719 with plasmid pBAD-nlpE	This study
ΔcpxR-pBAD	ΔcpxR with plasmid pBAD/Myc-His	This study
ΔcpxR-pnlpE	ΔcpxR with plasmid pBAD-nlpE	This study
DH5α	F-, *Δ(lacZYA*-*argF)U169, recA1, endA1, hsdR17(rk*−*, mk*+*), phoA, supE44, λ-*	TIANGEN
BL21 (DE3)	F-, *ompT, hsdS (r*_*B*_^−^ *m*_*B*_^−^*) gal, dcm* (DE3)	TIANGEN
Plasmids		
pET28a(+)	Kan, F1 origin, His tag	Novagen
pET28a-cpxR	pET28a(+) carrying *cpxR* gene	This study
pSTV28	Cm, p15A origin	Takara
pSTV28-cpxR	pSTV28 derivative harboring *cpxR* gene	This study
pBAD/Myc-His	Amp, ColE1 derivative cloning vector, pBAD (*ara*) promoter	Invitrogen
pBAD-nlpE	pBAD/Myc-His expressing *E. coli* K12 NlpE-His from the pBAD (*ara*) promoter	This study
pRCL	Cm, promoterless *lacZ*,	This study
pRCL-P_*hcp2B*_	pRCL harboring *hcp2B* promoter	This study
pKD46	Amp, expresses λ red recombinase	[45]
pKD3	Cm, template plasmid	[45]
pCP20	Cm, Amp, yeast Flp recombinase gene, FLP	[45]

### Construction of mutant and complemented strains

The *cpxR* gene deletion mutant was constructed using the Lambda Red recombinase system and appropriate primers (Table 2), as described previously [45] with some modifications. For complementation, the open reading frame and putative promoter of *cpxR* were subcloned into the low-copy plasmid pSTV28 (Takara, Dalian, China), which was then transformed into the mutant strain. The obtained mutant and complemented strains were validated by PCR and sequencing. For NlpE overexpression, the *nlpE* gene was cloned into the plasmid pBAD/Myc-His (Invitrogen, Carlsbad, CA, USA) and transformed into the APEC wild-type and mutant strains. To induce *nlpE* expression, we supplemented bacterial cultures with l-arabinose and performed appropriate antibiotic selection.

**Table 2:** **Primers used in this study**

Primers	Sequence (5′ to 3′)^a^	Target genes
For gene expression, deletion and complementation		
cpxREx-F	ACGGGATCCATGAATAAAATCCTGTTAGTTGATGA	*cpxR*
cpxREx-R	ACCAAGCTTTGAAGCAGAAACCATCAGATAG	*cpxR*
cpxRMu-F	GGATTAGCGACGTCTGATGACGTAATTTCTGCCTCGGAGGTATTTAAACAGTGTAGGCTGGAGCTGCTTC	Upstream region of *cpxR*
cpxRMu-R	AAGATGCGCGCGGTTAAGCTGCCTATCATGAAGCAGAAACCATCAGATAGCATATGAATATCCTCCTTAG	Downstream region of *cpxR*
cpxR-F	TTGATCTTCTGGACGACAGCA	*cpxR*
cpxR-R	CTCAGTACCGGTTAACTCCAGT	*cpxR*
cpxRCo-F	TCCGGATCCATATCAATAATTTCTTGCCGTTC	Upstream region of *cpxR*
cpxRCo-R	CCCAAGCTTGGCCTGACCAATAAAGTTACG	Downstream region of *cpxR*
nlpE-F	GAGCTCGAGGGTGAAAAAAGCGATAGTGA	*nlpE*
nlpE-R	TTCGAATTCTGCCCCAAACTACTGCAATC	*nlpE*
For RT-qPCR		
dnaE RT-F	ATGTCGGAGGCGTAAGGCT	*dnaE*
dnaE RT-R	TCCAGGGCGTCAGTAAACAA	*dnaE*
hcp2B RT-F	GTGAAATGCTGCCGAAAGTG	*hcp2B*
hcp2B RT-R	ACAATCGTCGCGTCAGTAAG	*hcp2B*
vgrG RT-F	CGAAGACGCAGATGACGATAC	*vgrG*
vgrG RT-R	GCGTGGATATAGACCTGTTCAC	*vgrG*
xmtU RT-F	GGTGTCATATCCGGTACATCTC	*xmtU*
xmtU RT-R	CTGAACCATGATAAGCAACAGG	*xmtU*
vipA RT-F	TAACACGCCGTTGGATGAG	*vipA*
vipA RT-R	GTTCAGCCGGAACAACAAAC	*vipA*
clpV RT-F	GAGACGCTCGCTACCATTATT	*clpV*
clpV RT-R	TGATTTCGTCCGTCACTTCC	*clpV*
hcp2A RT-F	ACGAAACCGGTGGACAAA	*hcp2A*
hcp2A RT-R	GGTTGGTGCGGTAGAATACA	*hcp2A*
For *lacZ* fusion and EMSA		
Phcp2B-F	TCTAAGCTTAGCTTATGTAATCGTGTTCTGAA	Upstream region of *hcp2B*
Phcp2B-R	GACGGATCCTTGAAATGTAACATGGGGTTGG	*hcp2B*
Phcp2BEMSA-F	AGCTTATGTAATCGTGTTCTG	Upstream region of *hcp2B*
Phcp2B EMSA-R	CATGGGGTTGGCATTTATGAA	*hcp2B*
Phcp2BdeletionEMSA-F	TTGACTAAAAATATATTTAAAC	Upstream region of *hcp2B*

^a^Restriction sites are underlined.

### Experimental animal infection

To determine the effect of CpxR on APEC virulence, we intramuscularly injected groups of eight 7-day-old ducks with bacterial suspensions containing 10^5^ Colony-Forming Units (CFUs). The number of CFUs in the injected inoculum was confirmed by plating on LB agar. Ducks inoculated with PBS were used as negative controls. Mortality was monitored daily until 7 days after infection.

The bacterial survival and competitive assays in vivo were measured as described previously [14, 46, 47]. Briefly, 7-day-old ducks were infected intratracheally with 10^8^ CFU bacterial suspensions with wild-type or mutant strains. Bacterial mixtures with equal amounts of wild-type and mutant strains were inoculated into ducks for the competitive assays. After 24 h, the ducks were euthanized and dissected, and the lung, liver and spleen were collected, weighed and homogenized. Serial dilutions of the homogenates were plated onto LB agar with or without chloramphenicol to distinguish the mutant strain or total bacterial loads. The competitive index (CI) was calculated for the mutant by dividing the output ratio (mutant/wild-type) by the input ratio (mutant/wild-type).

### Bacterial adhesion and invasion assays

Bacterial adhesion and invasion assays were performed as described previously [14, 47]. Chicken embryo fibroblast DF-1 cell monolayers were washed with Dulbecco’s modified Eagle’s medium (DMEM) without fetal bovine serum (Gibco, Grand Island, NY, USA) and infected with bacteria at a multiplicity of infection of 100 for 2 h at 37 °C under 5% CO_2_. After being washed with PBS, the cells were lysed with 0.5% Triton X-100, and the bacteria were counted by plating on LB agar plates. For invasion assays, the extracellular adherent bacteria were killed with DMEM containing 100 μg/mL gentamicin for 1 h, then washed and lysed with 0.5% Triton X-100 to enumerate the invasive bacteria.

### Bacterial competition assays in vitro

Bacterial competition assays were performed as described previously [11, 48] with some modifications. In brief, fresh donor and recipient strains were adjusted to an OD_600nm_ of 0.5 and mixed at a 5:1 ratio. This mixture was spotted on LB low-salt plates with nitrocellulose membranes for 6 h at 30 °C. Bacterial spots were collected, diluted and spotted onto LB plates with or without antibiotics for the selection of donor or recipient strains. Then the competition outcomes were calculated as the ratio of the donor strain to the recipient strain.

### Quantitative real-time reverse transcription PCR (qRT-PCR)

The expression of genes was investigated with qRT-PCR, as described previously [14, 46]. Briefly, total RNA was extracted from bacteria with TRIzol reagent (Invitrogen), and residual genomic DNA was removed with a Turbo DNase kit (Life Technologies, Carlsbad, CA, USA). cDNA synthesis was performed with a PrimeScript RT reagent kit (TaKaRa) according to the manufacturer’s protocol. qRT-PCR was conducted with SYBR Premix Ex Taq (TaKaRa) and gene-specific primers (Table 2), and the data were normalized to the expression of the housekeeping gene *dnaE*. The relative fold change was calculated via the ΔΔ*CT* method [49].

### Antibody production and Western blotting

Polyclonal anti-Hcp2B serum was raised in New Zealand white rabbits through subcutaneous immunization, as described previously [14]. For Western blotting, bacterial samples were subjected to sodium dodecyl sulfate–polyacrylamide gel electrophoresis and transferred onto a polyvinylidene fluoride membrane (Amersham Pharmacia Biotech, Piscataway, NJ, USA) as described previously [14, 47]. The proteins were reacted with the primary antibodies anti-Hcp2B or anti-DnaK (Enzo Life Sciences, Farmingdale, NY, USA), followed by horseradish peroxidase conjugated goat anti-mouse or goat anti-rabbit IgG secondary antibodies. The antigen–antibody complexes were visualized with chemiluminescence substrate (Amersham Pharmacia Biotech).

### β-Galactosidase assays

To confirm the promoter activity of the *hcp2B* gene, we performed β-galactosidase assays as described previously [50]. Briefly, the P_*hcp2B*_-*lacZ* fusions were constructed by cloning the P_*hcp2B*_ promoter into the promoterless plasmid pRCL. Then, the bacteria containing the P_*hcp2B*_-lacZ fusion plasmid or promoterless plasmid pRCL were collected and resuspended in Z buffer. The β-galactosidase activity was quantified with ortho-nitrophenyl-β-galactoside as the substrate. This assay was performed three times in triplicate.

### Electrophoretic mobility shift assay (EMSA)

The *cpxR* gene was cloned into the pET28a(+) plasmid (Novagen, Madison, WI, USA), and the recombinant proteins were expressed in *E. coli* BL21 (DE3) cells by addition of 1 mM isopropyl-β-d-thiogalactopyranoside. The purification of CpxR fusion protein was performed with a HisTrap high-performance column (GE Healthcare, Little Chalfont, Buckinghamshire, UK) as previously described [14]. The CpxR protein was phosphorylated with acetyl phosphate (Sigma, St. Louis, MO, USA) as previously described [51]. Then, EMSA were performed to determine the binding of phosphorylated CpxR (CpxR-P) to the *hcp2B* promoter. Briefly, the sequence of the *hcp2B* promoter region with or without the putative CpxR binding site was amplified and labeled with biotin. The biotin-labeled DNA probe (40 ng) was incubated with increasing concentrations of CpxR-P protein in EMSA binding buffer (10 mM Tris, 50 mM KCl, 5 mM MgCl_2_, 1 mM dithiothreitol, 0.1 mM MnCl_2_, 2.5% glycerol and 50 ng/μL poly[dI-dC]). After incubation for 30 min at room temperature, the reactions were subjected to electrophoresis and transferred to a nylon membrane. The biotin-labeled DNA was detected with a chemiluminescent substrate (Amersham Pharmacia Biotech). A competitive EMSA was performed by simultaneously incubating the biotin-labeled and unlabeled *hcp2B* promoter region with CpxR-P protein.

### Statistical analyses

Statistical analyses were conducted with the GraphPad Software package. One-way analysis of variance (ANOVA) was used to analyze the results of the adhesion, invasion and bacterial competition assays. Two-way ANOVA was used to analyze the qRT-PCR data. Analysis of the animal infection study results was performed with the non-parametric Mann–Whitney U-test. *P *< 0.05 was considered statistically significant.

## Results

### Deletion of *cpxR* attenuates APEC virulence in ducks

**Figure 1:**
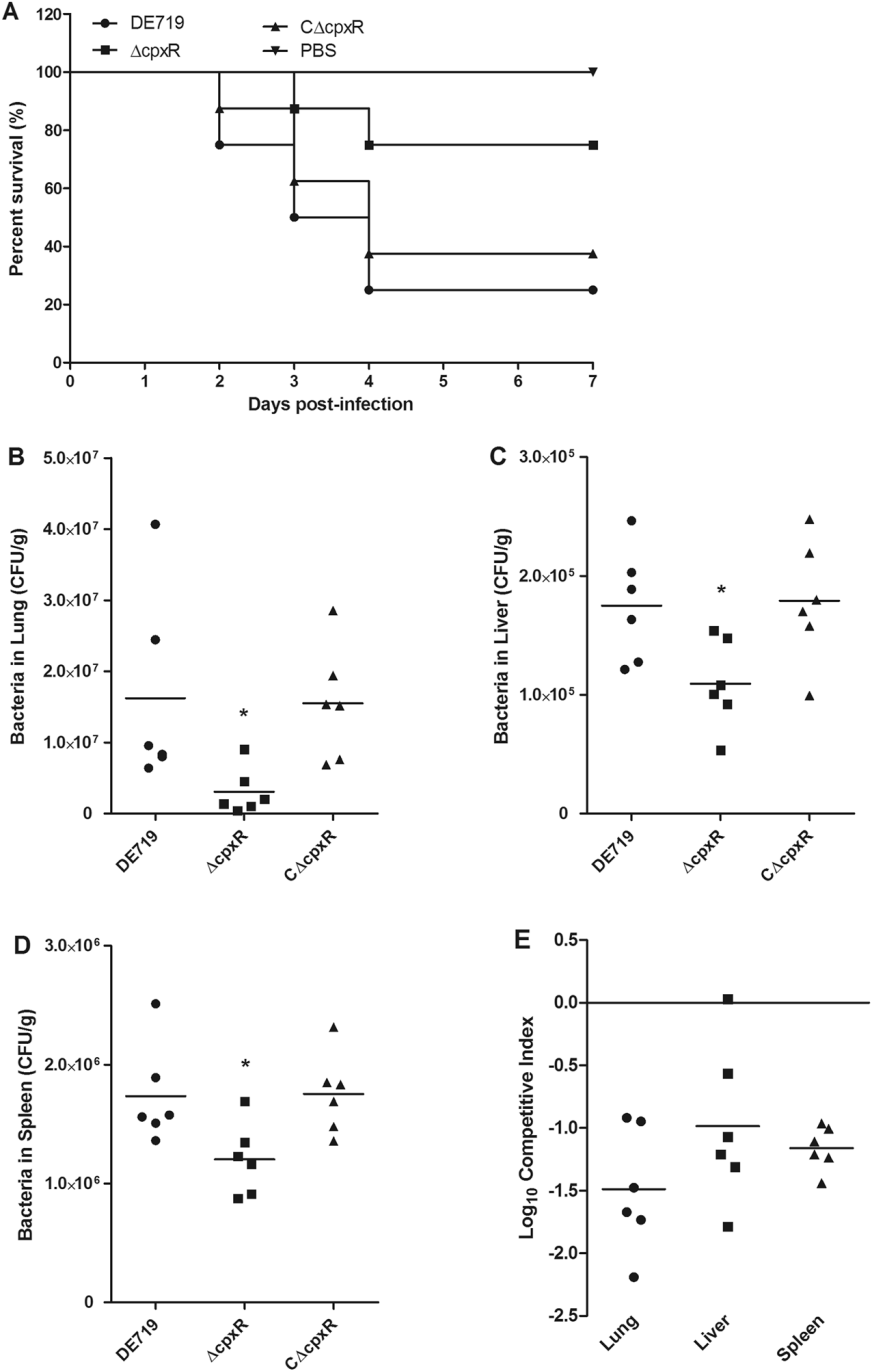
**CpxR is essential for efficient colonization and virulence of APEC. A** Determination of bacterial virulence. Seven-day-old ducks were infected with APEC strains, and the mortality was monitored until 7 days post-infection. Negative controls were injected with PBS. **B**–**E** Bacterial colonization, survival and competition in ducks. Seven-day-old ducks were infected with APEC strains in noncompetitive (**B**–**D**) and competitive (**E**) assays. Ducks were sacrificed at 24 h post-infection, and bacteria were recovered from the lungs, livers and spleens. The competitive indices were calculated and shown for the competitive assays (**E**). Nonparametric Mann–Whitney U-test was carried out to determine statistical significance (*P < 0.05).

The Cpx TCS has varying effects on the pathogenesis of different bacteria [26–35, 41, 44, 52, 53]. In APEC, the Cpx system has been implicated in the regulation of type 1 fimbriae and found to contribute to adherence, invasiveness and biofilm formation [41]. However, there is limited direct evidence that the CpxR affects the virulence of APEC in vivo. Thus, we constructed and characterized the Cpx TCS regulator encoding gene *cpxR* mutant and complemented strains. No significant differences were observed in the growth rate or the halo diameter between the wild-type and mutant strains. Then we compared the virulence of these APEC strains in a 7-day-old duck systemic infection model. The results showed that the mortality of DE719, ΔcpxR and CΔcpxR was 75% (6/8), 25% (2/8) and 62.5% (5/8), respectively. Moreover, the ducks infected with the mutant strain ΔcpxR died later than those infected with wild-type and complemented strains (Figure 1A). These results indicated that CpxR is required for full virulence APEC in ducks.

### CpxR provides the colonization and competition fitness for APEC during infection in vivo

To determine whether the decreased mortality was associated with altered bacterial colonization and survival capacity, we investigated the bacterial loads of APEC strains in the lung, liver and spleen at 24 h post-infection. The CFUs of the mutant strain ΔcpxR recovered from the lung, liver and spleen were significantly less than those of the wild-type and complemented strains in the noncompetitive assays (*P* < 0.05; Figures 1B–D). Bacterial pathogens compete with other bacteria, thus facilitating their replication and survival, and systemic infection [54]. Thus, the effects of CpxR on interbacterial competition fitness and pathogenesis in vivo were further examined by inoculation with equal amounts of mutant and wild-type strains. Similarly, the mutant strain ΔcpxR was strongly outcompeted by the wild-type strain in the tested organs (Figure 1E). These results indicated that CpxR potentiates the effective colonization and increases the competition fitness of APEC during infection in vivo.

### The role of CpxR in the invasion of APEC to DF-1 cells

**Figure 2:**
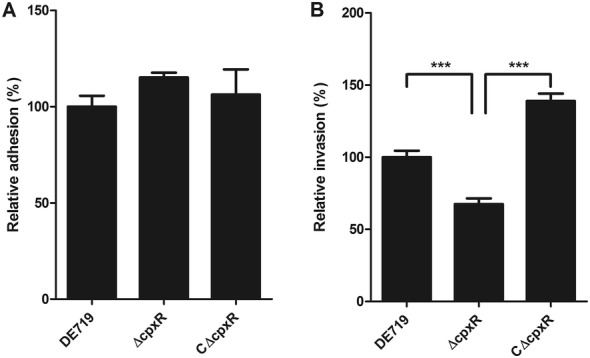
**CpxR contributes to the invasion of APEC to DF-1 cells.** The ability of APEC strains to adhere to (**A**) and invade into (**B**) DF-1 cells was compared. The mutant strain ΔcpxR showed significantly higher DF-1 cells invasion than the wild-type and complemented strains. Results are shown as relative adhesion and invasion capacity compared with those of the wild-type strain. Error bars indicate standard deviations. Statistical significance analysis was performed by using one-way ANOVA (***P < 0.001).

Bacterial adherence to and invasion of host cells are essential for effective colonization and pathogenesis. Moreover, CpxR is involved in the regulation of essential colonization and virulence factors. Therefore, we investigated the influence of CpxR on the bacterial adhesion to and invasion of DF-1 cells. Though the mutant strain ΔcpxR showed a slightly increased ability to adhere to DF-1 cells, no significant difference was found among these APEC strains (Figure 2A), in agreement with observations from a previous study [41]. However, the mutant strain showed a significantly lower ability than that of the wild-type strain (*P *< 0.001) to invade DF-1 cells. Furthermore, the defect in invasion capacity was restored via trans-complementation with the *cpxR* gene in the mutant strain (Figure 2B). These results suggested that CpxR contributes to the invasion of APEC to DF-1 cells.

### CpxR facilitates the interbacterial competition of APEC in vitro

**Figure 3:**
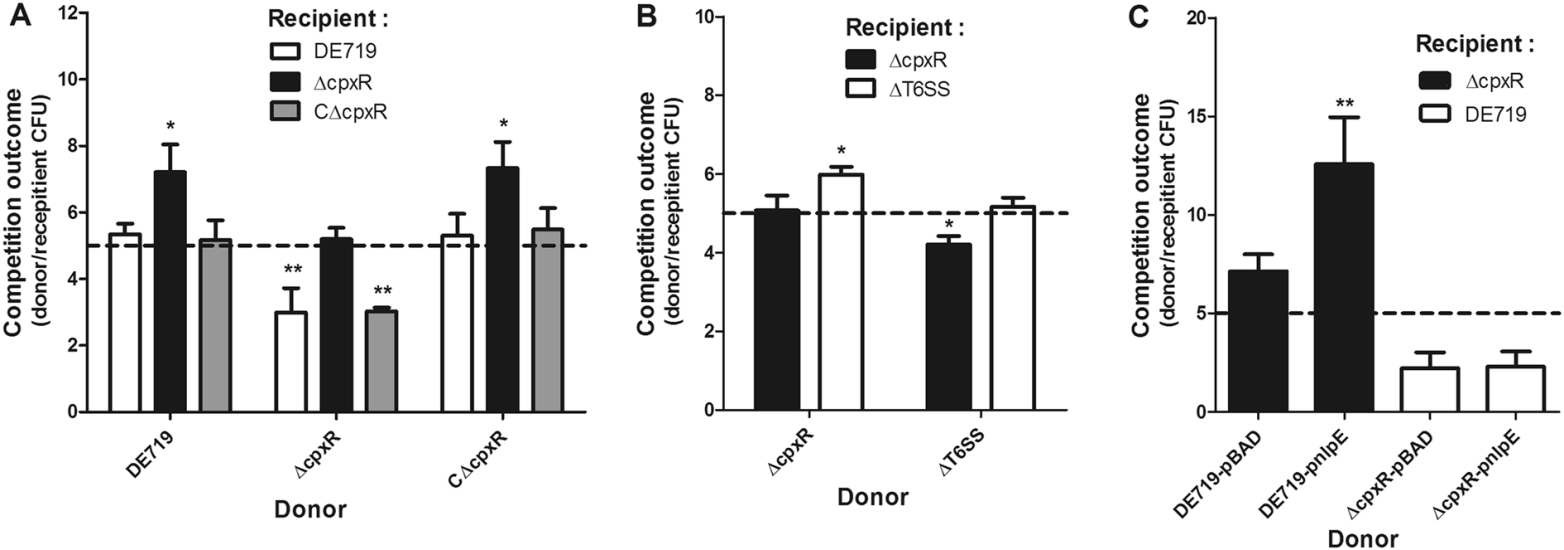
**CpxR facilitates interbacterial competition of APEC in vitro.** The indicated APEC donor and recipient strains were co-incubated at a ratio of 5:1, as shown by the dashed line. After incubation for 6 h at 30 °C, the donor and recipient strains were recovered. The competition outcomes between donor and recipient strains were calculated and are shown. **A**, **B** The interbacterial competition activities of wild-type, mutant and complemented strains were compared. **C** The interbacterial competition activity was also measured in the wild-type and mutant strains with NlpE overexpression. Statistical significance was assessed by using one-way ANOVA. (*P < 0.05; **P < 0.01).

To further investigate the role of CpxR in interbacterial competition, we co-cultured fresh APEC donor and recipient strains and counted the surviving bacteria. The survival of the recipient mutant strain ΔcpxR was significantly decreased when mixed with donor wild-type and complemented strains (*P *< 0.05). In contrast, the donor mutant strain ΔcpxR could not effectively kill the recipient wild-type and complemented strains (Figure 3A). Moreover, the mutant strain ΔcpxR exhibited growth comparable to that of the wild-type strain, thus indicating that the decreased interbacterial competition activity of the mutant strain ΔcpxR was unlikely to be due to a general growth defect. T6SSs have been found to confer a competitive advantage to bacteria, through their interbacterial activity. Previous studies by our group and others have indicated that APEC T6SS2 contributes to the invasion, survival, interbacterial competition and pathogenesis of APEC and NMEC [11, 14–16, 55]. Thus, a competition assay between the mutant strains ∆T6SS2 and ∆cpxR was performed. As expected, the strain ∆T6SS2 was outcompeted by the strain ΔcpxR, suggesting that the deletion of T6SS2 resulted in abolished interbacterial competition activity (Figure 3B).

Previous studies have indicated that overproduction of the outer membrane lipoprotein NlpE upregulates the Cpx pathway in *E. coli* [21, 22]. Thus, to further demonstrate the roles of CpxR in interbacterial competition, we overexpressed NlpE in wild-type and mutant strains, and measured the interbacterial competition activity of these strains. As expected, the overexpression of NlpE significantly increased the interbacterial competition activity in the wild-type strain (*P *< 0.01). However, no difference in interbacterial competition activity was observed for the mutant strain ΔcpxR overexpressing NlpE (Figure 3C). Collectively, these observations indicated that activation of CpxR facilitates the interbacterial competition activity of APEC.

### CpxR regulates the expression of T6SS2 in APEC

**Figure 4:**
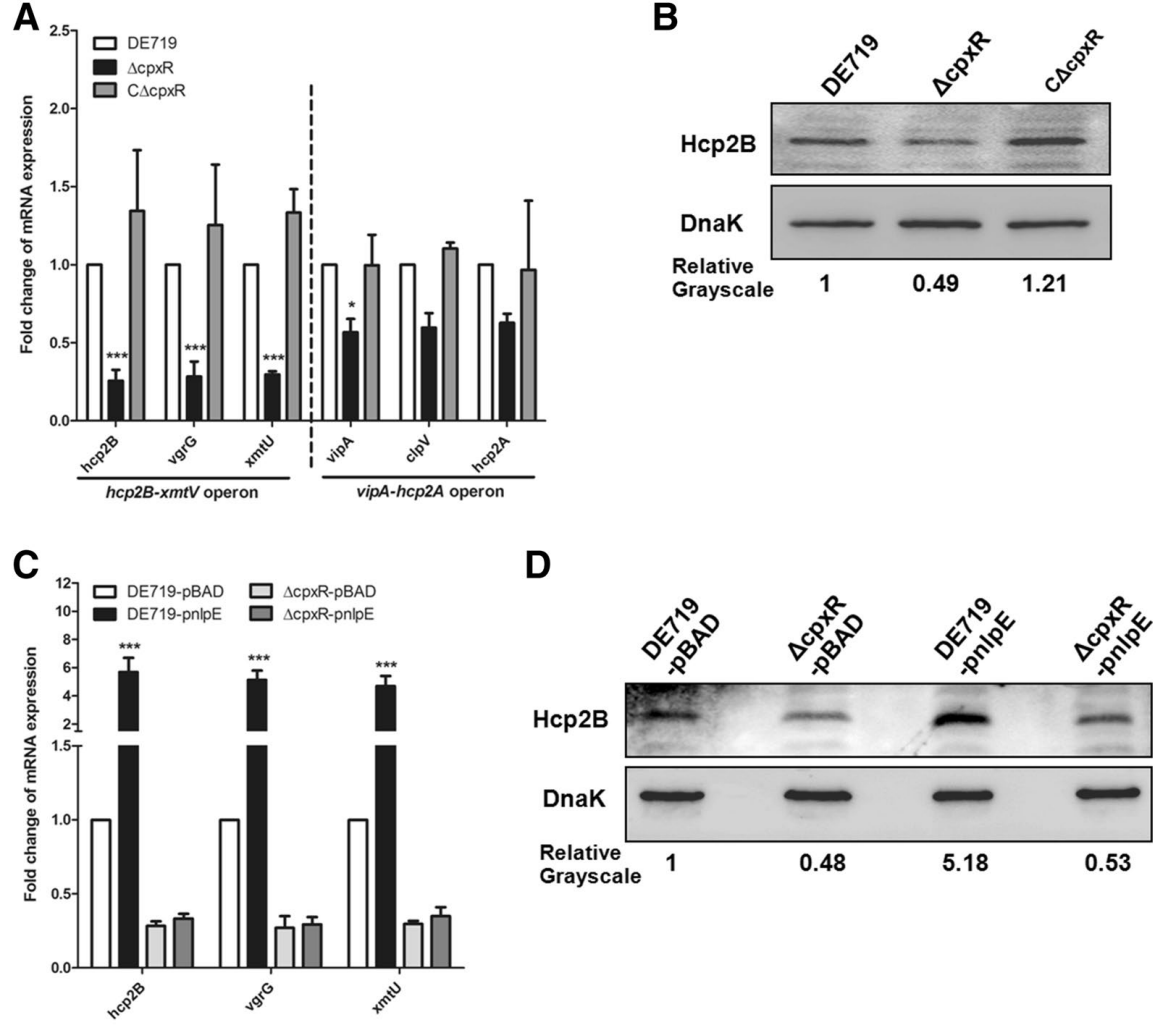
**CpxR regulates the expression of T6SS2 genes in APEC. A** The transcription levels of T6SS2 core genes were analyzed by qRT-PCR. **B** The levels of Hcp2B in APEC strains were confirmed with Western blotting. **C** qRT-PCR analysis for the transcriptional levels of the T6SS2 genes *hcp2B*, *vgrG* and *xmtU* in the absence or presence of NlpE in the wild-type and mutant strains. **D** The levels of Hcp2B in APEC strains with or without NlpE overexpression were determined by Western blotting. The qRT-PCR data are shown as relative expression ratios compared with that of the wild-type strain. For Western blotting, anti-DnaK antibody was used as a control. The expression of the Hcp2B protein was determined by quantifying the grayscale in Image J software. Two-way ANOVA was carried out to determine statistical significance (*P < 0.05; ***P < 0.001).

It has been shown that the Cpx TCS plays roles in regulating the T6SS in *Citrobacter rodentium* [36]. Hence, we sought to determine whether the decreased interbacterial activity of the mutant strain ΔcpxR was due to changes in T6SS2 expression. The expression of the T6SS2 core genes in these APEC strains was analyzed by qRT-PCR. The results showed that deletion of *cpxR* significantly downregulated the transcription of the *hcp2B* operon genes *hcp2B*, *vgrG* and *xmtU* (*P* < 0.001). However, the transcript levels of other T6SS core genes *vipA*, *clpV* and *hcp2A* changed only slightly (less than twofold) in the mutant strain ΔcpxR (Figure 4A), possibly because the *vipA* and *hcp2B* operons were transcribed in the opposite direction [11]. Additionally, we further validated the levels of Hcp2B via Western blotting. In agreement with the qRT-PCR results, Hcp2B production was decreased in the mutant strain ΔcpxR (Figure 4B). Moreover, complementation of the *cpxR* gene restored the transcription of T6SS2 genes (Figure 4A) and Hcp2B production (Figure 4B).

To further demonstrate that activation of CpxR promotes T6SS2 expression, we overexpressed NlpE in wild-type and mutant strains. The overexpression of NlpE significantly upregulated the transcription of *hcp2B*, *vgrG* and *xmtU* genes in the wild-type strain (*P* < 0.001) but not the mutant strain ΔcpxR (Figure 4C). Similarly, Hcp2B production increased with CpxR activation in the wild-type strain compared with the mutant strain ΔcpxR (Figure 4D). These data indicated that CpxR positively regulated the expression of T6SS2 *hcp2B* operon in APEC.

### CpxR protein directly binds the *hcp2B* promoter region

**Figure 5:**
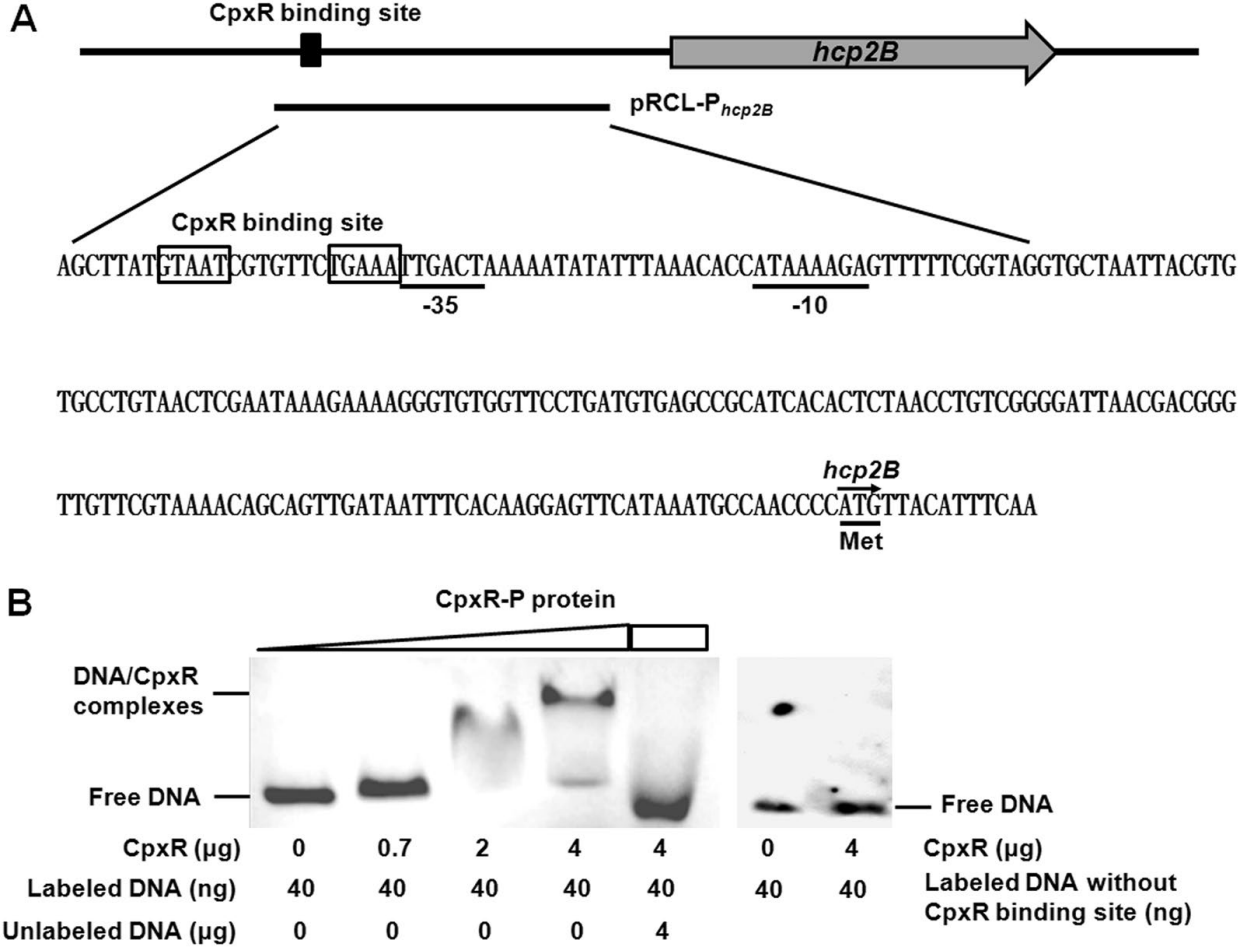
**CpxR directly binds the T6SS2** ***hcp2B*** **promoter region of APEC. A** The sequence and schematic representation of the *hcp2B* promoter region. The bold line shows the sequence used in the *lacZ* fusion and EMSA analyses. The putative CpxR binding site is indicated with boxes. The putative −35 and −10 elements of the promoter are indicated with underlining. **B** EMSA for the binding of CpxR-P protein to the *hcp2B* promoter region. The *hcp2B* promoter DNA fragment with or without the CpxR binding site was amplified and biotin-labeled, and the biotin-labeled probe was mixed with increasing amounts of CpxR-P protein. For the specific competitive EMSA, CpxR-P protein was incubated with both the biotin-labeled and the unlabeled DNA probes. The biotin-labeled DNA was detected with a chemiluminescent substrate. The concentrations of CpxR-P protein and probes are shown below the figure.

To investigate whether CpxR directly regulates *hcp2B* operon expression, we first searched for a putative CpxR binding site in the promoter sequence of the *hcp2B* gene according to the consensus sequence CpxR binding site [GTAAA(N)_4–8_GTAAA] reported in *E. coli* [56, 57]. One putative CpxR binding site was found to be located in the promoter region of the *hcp2B* gene (Figure 5A), thus suggesting that CpxR might directly regulate the *hcp2B* operon. Furthermore, we demonstrated that the *lacZ* transcriptional reporter fusion P_*hcp2B*_-*lacZ* under the control of the *hcp2B* promoter showed β-galactosidase activity (Additional file 1). Next, the *hcp2B* promoter sequence including the potential CpxR binding site was amplified, biotin-labeled and subjected to EMSA analysis, to verify the direct binding of CpxR-P to the *hcp2B* promoter region. Indeed, the migration of the *hcp2B* probe was slowed in the presence of increasing amounts of CpxR-P. In addition, binding specificity was confirmed via a competitive EMSA, and the binding of CpxR-P to biotin-labeled probes was abolished by an excess of specific competitor consisting of unlabeled *hcp2B* promoter fragments. In addition, the *hcp2B* promoter fragments without the CpxR binding site were no longer shifted by the CpxR-P protein (Figure 5B). Taken together, these results suggested that CpxR directly binds the T6SS2 *hcp2B* operon in APEC.

## Discussion

Bacterial virulence is determined by the expression of virulence factors, and pathogens must be able to precisely modulate gene expression to facilitate their adaptation and survival in the local microenvironment or host cells. The infection process requires rapid adaptation to the host environment through various regulatory mechanisms. TCSs are activated in response to changing environmental cues, thus representing one mechanism enabling bacterial adaptation through transcriptional regulation of gene expression [18, 19]. The Cpx TCS responds to cell envelope stress, which is used by bacteria to maintain cell envelope integrity [23, 58]. Increasing studies implicate the Cpx TCS in the virulence of numerous Gram-negative bacteria [26–35, 41, 44, 52, 53]. These findings prompted us to determine the regulatory roles of the Cpx TCS in the fitness and virulence of APEC. This study provided evidence that the regulator CpxR contributes to the interbacterial competition activity, survival and virulence of APEC at least through directly regulating the expression and function of T6SS2.

By using a duck systemic infection model, we demonstrated that inactivation of the *cpxR* gene attenuates the virulence of APEC. Moreover, the complemented strain showed recovered virulence. Thus, we concluded that CpxR is necessary for full virulence of APEC. APEC initially infects poultry via the respiratory tract, then spreads systemically throughout the entire body, thus resulting in subsequent bacteremia and death. Colonization and invasion are important virulence parameters in APEC infection [6, 59]. A previous study has indicated that the Cpx TCS affects virulence features including the adherence, invasiveness, motility and biofilm formation of APEC through the direct binding of CpxR-P to the *fimA* promoter [41]. Indeed, type 1 fimbriae are filamentous surface organelles known to contribute to adherence, invasiveness and biofilm formation [6, 60]. Our results indicated that inactivation of CpxR does not affect the adherence ability of APEC, in agreement with findings from a previous study. This observation might have been because CpxR affects the ON/OFF orientation on the *fimA* promoter [41]. In contrast, the mutant strain ΔcpxR showed a significantly decreased ability to invade into host cells. The decreased capacity of the mutant strain to invade DF-1 cells might contribute to the inability of this mutant to effectively colonize, survive in and infect ducks. Similarly, decreased colonization and virulence have been observed for the *cpx* mutant strain in different Gram-negative pathogens [31, 61–63].

Pathogens have developed diverse attack strategies to efficiently compete with other bacteria for limited resources and facilitate survival and infection [13]. Our results showed that the interbacterial competition capacity of the mutant strain ΔcpxR was much lower than that of the wild-type strain in vitro. Moreover, the mutant strain ΔcpxR was significantly outcompeted by the wild-type strain in ducks. These results indicated that CpxR contributes to competition and survival in vivo, and might be responsible for the systemic infection and virulence of APEC. Consistently with these results, CpxRA influences the initial colonization and outgrowth of *Xenorhabdus nematophila* during infection through regulation of the *nil* locus [62]. In addition, the Cpx system may affect bacterial virulence by modulating surface characteristics and serum resistance of pathogens [61].

The *E. coli* CpxR regulon contains hundreds of genes, according to genome-wide screens for the putative CpxR binding sequence [56, 57]. In addition to regulating protein folding and degradation, CpxR also plays roles in regulating T6SS expression in *Citrobacter rodentium* [36]. T6SSs are a common strategy used by many pathogens to mediate successful infections in hosts [7–14, 16]. The APEC T6SS2 functions in the invasion, survival, interbacterial competition and pathogenesis of APEC and NMEC [11, 14–16, 55]. In this study, we observed that CpxR influenced the expression of T6SS2; we then sought to investigate the mechanism of CpxR mediated T6SS2 regulation in APEC. We found that inactivation of the regulator CpxR significantly downregulated the transcription of the T6SS2 *hcp2B* operon. Although the fold-change in the Hcp2B protein was less than the changes at the transcription level, it was also decreased in the mutant strain ΔcpxR. Previous study showed that the *hcp2B* operon is necessary for the interbacterial competition activity of T6SS2 in APEC [11]. Moreover, our results showed that the decreased expression of Hcp2B protein was in good agreement with the interbacterial competition activity for mutant strain ∆cpxR. Our previous study has also found different fold-changes in the transcription and expression levels [64], possibly because many factors affect protein expression. Collectively, these results suggested that CpxR promotes the expression of APEC T6SS2. This result is consistent with the regulatory role in *Citrobacter rodentium* [36]. Additional, the decreased expression of the T6SS2 *hcp2B* operon, including the interbacterial effector encoding the gene *xmtU*, might be responsible for the decreased invasion, diminished survival and impaired competition of the mutant strain ΔcpxR. Many environmental conditions upregulate the Cpx pathway. The lipoprotein NlpE has been shown to activate the Cpx pathway when it is overproduced [21–23, 58, 65, 66]. We observed that NlpE overexpression in the wild-type strain DE719 increased the T6SS2 *hcp2B* operon expression and subsequently significantly enhanced interbacterial competition. In contrast, no regulatory effect was found for the mutant strain ΔcpxR overexpressing NlpE. An in silico analysis identified a putative CpxR binding site in the *hcp2B* promoter region. Furthermore, we provided evidence that CpxR-P binds the promoter region of the *hcp2B* operon, thus suggesting that CpxR directly regulates T6SS2 *hcp2B* expression. Taken together, these results indicated that the regulation of T6SS2 expression and function by CpxR may contribute to the infection and pathogenesis of APEC.

**Figure 6:**
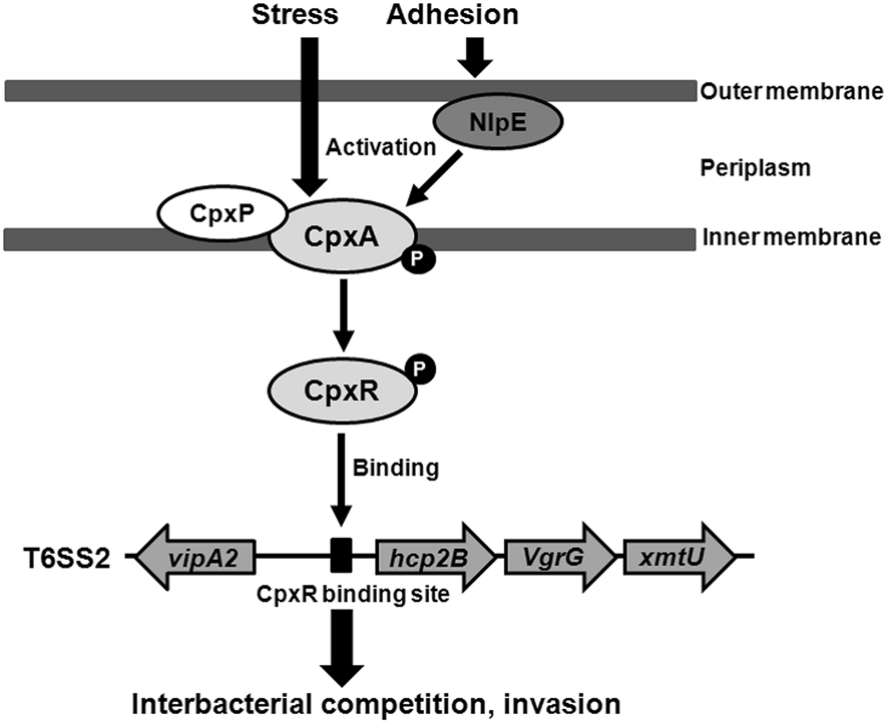
**Schematic diagram of CpxR-mediated regulation of T6SS2 expression in APEC.** According to previous studies, many environmental stresses and surface adhesion sensed by the NlpE protein can activate the Cpx response [21, 22]. After activation, the CpxA protein phosphorylates the CpxR protein. The phosphorylation of CpxR enhances its binding to the T6SS2 *hcp2B* promoter and consequently upregulates transcription of the *hcp2B* operon. The upregulation of T6SS2 contributes to increased antibacterial competition, invasion and survival, which are required for the virulence of APEC.

In conclusion, we provided evidence that CpxR contributes to colonization, invasion and interbacterial competition fitness for APEC in vivo and in vitro. The most provocative finding of this study is that CpxR may contribute to APEC virulence by directly binding and regulating the expression and function of T6SS2 *hcp2B* (Figure 6). Together with the conclusion that the Cpx TCS regulates type 1 fimbriae-mediated virulence features, including adherence, motility and biofilm formation of APEC [41], these compelling findings suggest that the Cpx TCS controls the virulence of APEC via various regulatory mechanisms. Thus, further studies are needed to fully understand the regulatory network through which Cpx TCS contributes to virulence.

## Additional file

**Additional file 1.** **The β-galactosidase activity of the** ***lacZ*** **transcriptional reporter fusion P**_***hcp2B***_ **-** ***lacZ*** **was measured as described in “** **Materials and methods** **”.**
