# Supplementary figures and images for: The CpxR regulates type VI secretion system 2 expression and facilitates the interbacterial competition activity and virulence of avian pathogenic Escherichia coli
Source: Vet Res. 2019 May 24;50:40. doi: 10.1186/s13567-019-0658-7 (PMC6534853; doi:10.1186/s13567-019-0658-7)

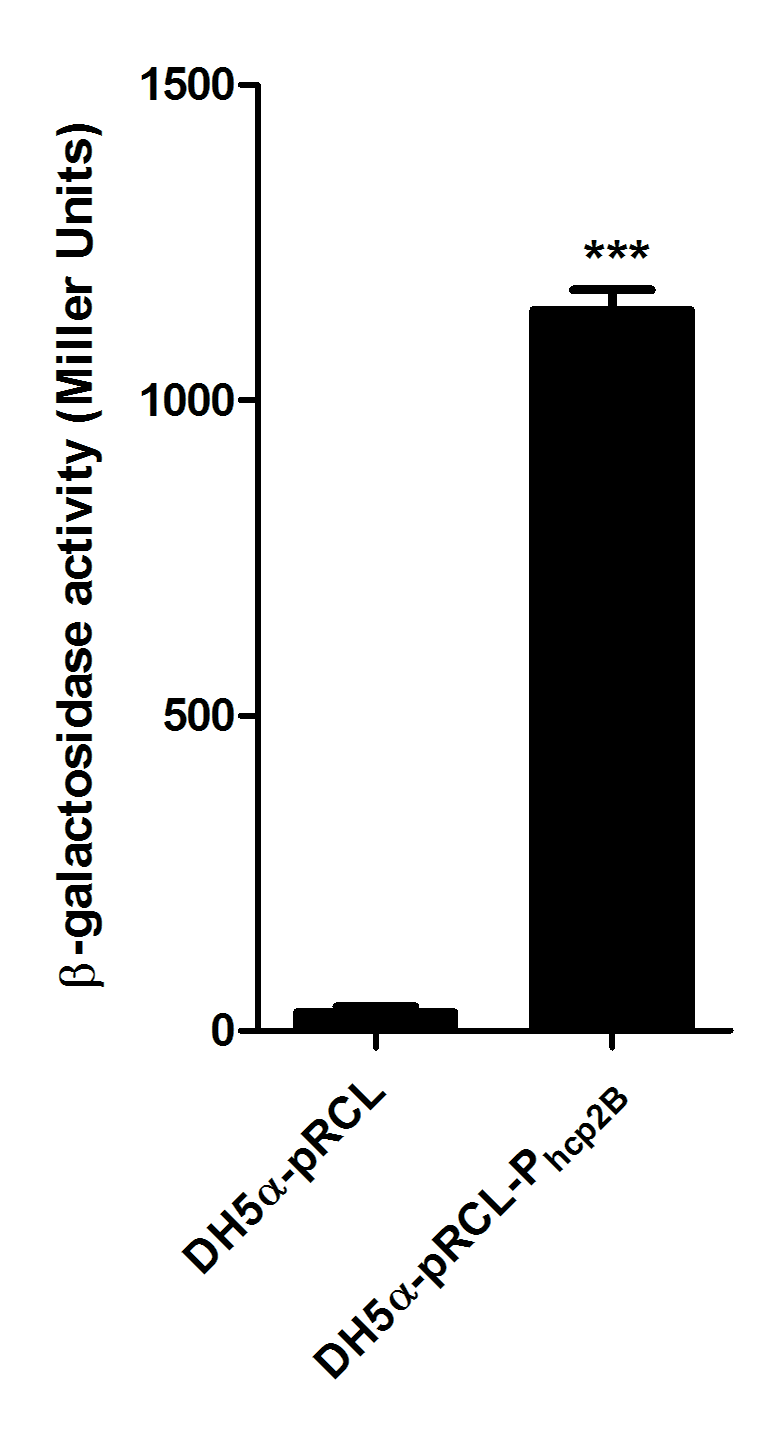

Supplement: Supplementary file 1 — Additional file 1. The β-galactosidase activity of the lacZ transcriptional reporter fusion P hcp2B - lacZ was measured as described in “ Materials and methods ”. [file 13567_2019_658_MOESM1_ESM.docx]
